# Factors associated with parental reasons for “no-intent” to vaccinate female adolescents with human papillomavirus vaccine: National Immunization Survey - Teen 2008–2012

**DOI:** 10.1186/s12887-017-0804-1

**Published:** 2017-02-13

**Authors:** Vinay K. Cheruvu, Madhav P. Bhatta, Lauren N. Drinkard

**Affiliations:** Department of Biostatistics, Environmental Health Sciences, and Epidemiology, College of Public Health, Kent State University, Hilltop Drive, 320 Lowry Hall, Kent, 44242 OH USA

**Keywords:** HPV vaccine, Unvaccinated female adolescents, Reasons for no-intent, National Immunization Survey - Teen

## Abstract

**Background:**

1) To identify socio-demographic factors associated with parental “no-intent” for their 13–17 year old unvaccinated daughter to receive the human papillomavirus (HPV) vaccine series within the next twelve months, 2) to describe patterns in “no-intent” by socio-demographic factors, and 3) to identify socio-demographic factors associated with parental reasons for “no-intent”.

**Methods:**

Data from 2008–2012 National Immunization Survey - Teen (NIS - Teen) were examined in this study. Parents with “no-intent” to vaccinate their daughters were asked to identify reasons for their decision. All responses were categorized into five domains identified as barriers to receive the HPV vaccine series: *1) Safety and Effectiveness Concerns*; *2) Systemic Barriers*; *3) Vaccine Misinformation; 4) Lack of Knowledge about the Vaccine*; and *5) Socio-cultural Barriers*. Multivariable logistic regression models were performed to address the study objectives.

**Results:**

Number of people in the household, household income, mother’s age, education, health insurance, recommendation of a health care provider, and the survey year were significantly associated with parental “no-intent”. Race/ethnicity, mother’s education, marital status, recommendation of a health care provider, household income, age of the unvaccinated daughter, and the survey year, were significantly associated with one or more domains identified as barriers to receive the HPV vaccine.

**Conclusions:**

This study identified sub-groups of parents across different socio-demographic factors with “no-intent” for their adolescent daughters to receive the HPV vaccine. Developing strategies that target educational tools towards the identified sub-groups of parents about the purpose, safety, and efficacy of the HPV vaccine, and HPV infection, may help increase HPV vaccine acceptance, initiation and completion rates.

## Background

In the United States (U.S.), human papillomavirus (HPV) is the most prevalent sexually transmitted infection (STI) with up to 24.5% HPV infection among females aged 14–19 years [1–3]. HPV types 6 and 11, cause 90% of genital warts and HPV types 16 and 18, cause 70% of cervical cancers [4]. Oncogenic HPV also causes vulvar, vaginal, anal cancers [5, 6]. In 2012, there were two highly efficacious HPV vaccines on the market licensed by The Food and Drug Administration (FDA); the bivalent vaccine (Ceravix) which provides protection against high-risk oncogenic HPV types 16 and 18 only, and the quadrivalent vaccine (Gardasil) which provides protection against types 16 and 18, as well as two low risk types 6 and 11 [7–9]. In 2014, the 9-valent (Gardasil 9) virus-like particle HPV vaccine was approved which has the potential to increase cervical cancer prevention from 70% to 90% [10, 11]. The Healthy People 2020 goal for HPV vaccine coverage among female adolescents aged 13–15 years in the U.S. is 80% [12]. Among females aged 14–19 years, Markowtiz and colleagues reported a decline in the prevalence of vaccine preventable HPV infections from 11.5% in 2003–2006 (the pre-vaccine era) to 5.1% in 2007–2010 (the post-vaccine era) [13]. Despite a significant increase in HPV vaccination coverage during 2007–2011, the increase in the percentage of unvaccinated girls with at least one missed opportunity for HPV vaccination from 20.8% to 84.0% is a major public health concern [14]. These numbers highlight the potential of the HPV vaccine, the importance of developing educational strategies for parents about the vaccine, and the need for targeting vaccination campaigns towards adolescents to reduce HPV infections and their sequelae, most notably genital warts and cervical cancer.

A considerable number of studies have focused on the factors associated with initiation and completion of the HPV vaccine series [15–30]. For example, two studies reported older age of adolescent females was associated with initiation and completion of HPV vaccination [22, 23]. Only recently, the research focus has shifted to understand the population of unvaccinated teens, factors associated with “intent”, and parental reasons for “no-intent” for their daughters to receive the HPV vaccine [25–35]. However, such studies were limited in that they only reported the most common reasons cited by parents for “no-intent” to vaccinate their adolescents, while failing to identify the underlying socio-demographic characteristics of such parents. Parental attitudes and beliefs regarding vaccines in general and the HPV vaccine in particular, affect the likelihood that their children will receive vaccines [36]. It is particularly important that we identify these socio-demographic sub-groups and develop targeted strategies to improve acceptance and uptake of the HPV vaccine.

In a recent study among unvaccinated females aged 15–25 years, Liddon and colleagues described the correlates of the main reasons for foregoing vaccination in females [33]. However, to our knowledge, factors associated with various self-disclosed parental reasons for “no-intent” to vaccinate their daughters have not been studied in a nationally representative sample of unvaccinated female adolescents aged 13–17 years.

In this study, we used a sub-sample of unvaccinated females aged 13–17 years from the National Immunization Survey - Teen (NIS - Teen), 2008–2012, to address three specific objectives: 1) To identify socio-demographic factors associated with parental “no-intent” for their 13–17 year old unvaccinated daughter to receive the human papillomavirus (HPV) vaccine series in the next twelve months, 2) to describe patterns in “no-intent” by socio-demographic factors, and 3) to identify socio-demographic factors associated with parental reasons for “no-intent”.

## Methods

### National Immunization Survey-Teen (NIS-Teen)

Data from 2008–2012 NIS-Teen surveys were used for the analyses [37]. The NIS-Teen survey is implemented annually by the National Center for Immunization and Respiratory Diseases and the National Center for Health Statistics of the Centers for Disease Control and Prevention. It represents a stratified national probability sample of households in the U.S. that include all 50 states, the District of Columbia, and the U.S. Virgin Islands. Details about methodology and weighting procedures, have been previously published [38]. All estimates were based on adolescents with adequate provider data. The observations from the U.S. Virgin Islands were excluded from all analyses. The final sample size was 23,722. This study was exempt from review by Kent State University’s IRB because NIS - Teen data are publicly available and deidentified.

### No-intent to receive the HPV vaccine series

Parent (or guardian) of a female adolescent with no history of HPV vaccination at the time of the interview was asked the question “*how likely is it the teen will receive HPV vaccinations in the next 12 months?”*. Of the five possible responses, parents who responded “*very likely”* or “*somewhat likely”* were considered as having “intent” and those who responded “*not too likely”*, “*not likely at all”*, or “*not sure/do not know”* were considered as having “no-intent” for their daughter to receive the HPV vaccine series within the next 12 months. The percentage of parents who responded “*not sure/do not know”* was very small (<3%). Parents with “no-intent” were then asked the question, “*What is the main reason* [*your teen*] *will not receive HPV shots in the next 12 months?* “ Parents were allowed to report one or more reasons from a list which CDC created. For the present analyses, we created the following five domains for the reasons parents reported: i) ***Safety and Effectiveness Concerns***, ii) ***Systemic Barriers***, iii) ***Vaccine Misinformation***, iv) ***Lack of Knowledge about the Vaccine***, and v) ***Socio-cultural Barriers***. Table 1 shows the questions that went into each domain created based on qualitative groupings. Each domain is treated as a binary outcome in our analyses: “Yes” if a parent reported at least one of the reasons and “No” if no reason was reported in the corresponding domains.

**Table 1:** Reasons for no-intent to receive human papillomavirus vaccine (HPV) series among unvaccinated female adolescents aged 13–17 years, National Immunization Survey - Teen, 2008-2012

Domain	Reason #	Main reason teen will not receive HPV shots in the next 12 months
Safety and Effectiveness Concerns	HPVI_REAS_11	Safety Concern/Side Effects
	HPVI_REAS_12	Effectiveness Concern
	HPVI_REAS_21	More info/New Vaccine
Systemic Barriers	HPVI_REAS_1	Not Recommended
	HPVI_REAS_10	Costs
	HPVI_REAS_23	Not Available
	HPVI_REAS_24	Not a School Requirement
	HPVI_REAS_26	No OB/GYN
	HPVI_REAS_28	No Doctor or Doctor’s Visit Not Scheduled
Vaccine Misinformation	HPVI_REAS_2	Not Needed or Not Necessary
	HPVI_REAS_5	Not Sexually Active
	HPVI_REAS_6	Not Appropriate Age
	HPVI_REAS_25	Increased Sexually Activity Concern
Lack of Knowledge about the Vaccine	HPVI_REAS_3	Lack of Knowledge
Socio-cultural reasons	HPVI_REAS_16	Don’t Believe in Immunizations
	HPVI_REAS_17	Family/Parental Decision
	HPVI_REAS_19	Religion/Orthodox

### Statistical analysis

Of the 23,722 unvaccinated females (2008: *n* = 5,126; 2009: *n* = 4,901; 2010: *n* = 4,614; 2011: *n* = 5,231; and 2012: *n* = 3,850), 21,467 had information on all the covariates of interest. Of these, parents of 12,716 females were in the “no-intent” group, of which 12,274 of them reported at least one reason for their decision.

The unadjusted weighted prevalence estimates with 95% confidence intervals (CIs) were computed to describe the socio-demographic characteristics of unvaccinated female adolescents. Univariate analyses were performed to test for associations between socio-demographic factors and parental “intent” vs. “no-intent” for their daughters to receive the HPV vaccine series within the next 12 months. Multivariable logistic regression analysis was performed to determine the factors associated with “no-intent”. To describe how the socio-demographic factors associated with parental “no-intent” changed over time, we computed the unadjusted weighted prevalence estimates with 95% CIs for each survey year. The sample size for these analyses was 21,467. To determine how the socio-demographic factors associated with parental reasons for “no-intent”, we performed multivariable logistic regression analysis treating the five domains as binary outcomes (Yes/No) correspondingly. The sample size for these analyses was 12,274.

Sampling weights provided in the 2008–2012 NIS - Teen public use data were used to obtain population-based estimates. All analyses were performed using SAS® version 9.3 (Cary, NC, USA) survey procedures (PROC SURVEYFREQ, PROC SURVEYMEANS, PROC SURVEYLOGISTIC) to account for the complex sampling design of the NIS-Teen data. All analyses were conducted in 2014.

## Results

### Sample characteristics

Overall, 51.1% (95% CI: 50.3%–52.0%) of the female adolescents were completely unvaccinated for HPV [2008: 61.0% (95% CI: 58.9%–63.0%) ; 2009: 53.9% (95% CI: 52.0%–55.7.0%) ; 2010: 52.0% (95% CI: 50.1%–53.8%) ; 2011: 46.8% (95% CI: 45.1%–48.4%) ; 2012: 42.0% (95% CI: 40.1%–43.7%)]. The mean age of these adolescents was 14.3 years. Table 2 presents the socio-demographic characteristics of the unvaccinated adolescent females in this study. Sixty percent were White non-Hispanic, 71.6% of mothers of unvaccinated females were married, 33.6% were college graduates, and 46.6% of were between the ages 35–44. Sixty-three percent reported that a healthcare provider had not recommended that their daughter receive the HPV vaccine series.

**Table 2:** Sample characteristics of unvaccinated^a^ female adolescents aged 13-17 years against human papillomavirus (HPV), National Immunization Survey - Teen, 2008 – 2012 (*n* = 21,467)

Characteristics	*n*	Weighted % or median estimate	95% confidence interval
Adolescent age, years			
13	5,105	22.9	(21. 9–23.9)
14	4,722	21.1	(20.1–22.1)
15	4,256	20.7	(19.7–21.8)
16	3,936	19.2	18.2–20.3)
17	3,448	16.1	(15.2–16.9)
Race/ethnicity			
White, non-Hispanic	15, 036	60.1	(58.9–61.4)
Black, non-Hispanic	2,385	15.5	(14.5–16.4)
Hispanic	2,393	16.8	(15.7–17.9)
Other^b^	1,663	7.6	(6.9–8.3)
Number of people in the household	21,467	3.8	(3.7–3.8)
Annual household income, US dollars			
≤35,000	4,973	30.8	(29.5–32.0)
35,001–75,000	7,055	32.5	(31.3–33.7)
>75,000	9,439	36.7	(35.6–37.9)
Mother’s education			
Less than high school	1,859	12.9	(11.9–14.0)
High school	4,321	26.9	(25.5–27.9)
Some college	6,393	26.8	(25.6–27.8)
College graduate	8,894	33.6	(32.5–34.7)
Mother’s marital status			
Married	16,518	71.6	(70.4–72.8)
Other^c^	4,949	28.4	(27.2–29.6)
Mother’s age, years			
≤34	1,518	8.9	(8.1–9.7)
35–44	9,263	46.6	45.4–47.9)
≥ 45	10,686	44.4	(43.2–45.7)
Health insurance status^d^			
Private	14,916	63.5	(62.20–64.8)
SCHIP or Medicaid	3,732	22.5	(21.4–23.7)
IHS or Military	347	1.2	(1.0–1.3)
Other	1,123	4.8	(4.2–5.4)
Uninsured	1,349	8.0	(7.3–8.8)
Healthcare provider recommended the HPV vaccine			
No	12, 952	63.1	(61.9–64.3)
Yes	8,515	36.9	(35.7–38.1)
Survey year			
2008	4,605	24.0	(22.9–25.2)
2009	4,424	21.0	(20.0–22.0)
2010	4,173	20.0	(19.0–21.0)
2011	4,761	18.4	(17.5–19.3)
2012	3,504	16.6	(15.7–17.5)

^a^Has had zero doses of HPV vaccine

^b^Includes non-Hispanic other racial/ethnic groups and biracial

^c^Never married/widowed/divorced/separated

^d^SCHIP = State Children’s Health Insurance Program; IHS = Indian Health Service

### Socio-demographic factors associated with no-intent to receive the HPV vaccine series

During 2008–2012, 59.9% (95% CI: 58.7%–61.1%) of parents of unvaccinated adolescent females indicated that they did not intend to have their daughters vaccinated against HPV within the next 12 months. Table 3 describes the socio-demographic characteristics of unvaccinated females across “no-intent” vs. “intent” groups and presents the factors significantly associated with parents’ “no-intent” to vaccinate their daughters: number of people in the household (Adjusted Odds Ratio: AOR: 1.05 [95% CI: 1.01–1.10]); annual household income of $35,001–$75,000 (AOR: 1.21 [95% CI: 1.02–1.44]) compared to ≤ $35,000; mothers with some college education (AOR: 1.44 [95% CI: 1.16–1.79]) or who are college graduates (AOR: 1.28 [95% CI: 1.03–1.60]) compared to mothers with less than high school education ; mothers ≥45 years of age (AOR: 1.32 [95% CI: 1.06–1.65]) compared to younger mothers (≤34 years); “Other” insurance (AOR: 1.36 [95% CI: 1.05–1.75]) compared to private insurance; no recommendation from a health care provider (AOR: 2.02 [95% CI: 1.82–2.53]) compared to recommendation from a health care provider; and the survey year, 2009 (AOR: 1.30 [95% CI: 1.10–1.52]), 2010 (AOR: 1.57 [95% CI: 1.33–1.86]), 2011 (AOR: 1.25 [95% CI: 1.06–1.47]), and 2012 (AOR: 1.26 [95% CI: 1.06–1.50]) compared to survey year 2008. Parents who identified as Hispanics were significantly less likely to report no-intent to vaccinate their daughters (AOR: 0.79 [95% CI: 0.66–0.95]) compared to White, non-Hispanics.

**Table 3:** Factors associated with no-intent to receive^a^ the human papillomavirus vaccine (HPV) series among unvaccinated female adolescents aged 13–17 years, 2008–2012 national immunization survey—teen (*n* = 21,467)

Characteristics	Unadjusted weighted proportion (95% CI) or median		Adjusted odds ratio (95% confidence interval)
	No-intent to initiate (*n* = 12,716)	Intent-to-initiate (*n* = 8,751)	
Adolescent age, years			
13	23.0 (21.7–24.4)	22.8 (21.2–24.3)	1.00
14	20.9 (19.6–22.2)	21.3 (19.8–22.9)	0.96 (0.83–1.12)
15	19.9 (18.6–21.2)	22.0 (20.2–23.7)	0.88 (0.75–1.03)
16	20.0 (18.6–21.5)	18.0 (16.5–19.6)	1.06 (0.90–1.25)
17	16.1 (15.1–17.2)	15.9 (14.5–17.3)	0.93 (0.79–1.10)
Race/ethnicity of teen			
White, non-Hispanic	62.0 (60.3–63.6)	57.4 (55.4–59.4)	1.00
Black, non-Hispanic	15.3 (14.1–16.4)	15.8 (14.2–17.3)	0.96 (0.81–1.12)
Hispanic	15.2 (13.8–16.6)	19.3 (17.5–21.1)	0.79 (0.66–0.95)
Other^b^	7.6 (6.8–8.5)	7.6 (6.3–8.9)	0.96 (0.76–1.22)
Number of people in the household per person over two	3.79 (3.74–3.84)	3.75 (3.69–3.81)	1.05 (1.01–1.10)
Annual household, US dollars			
≤35,000	28.4 (26.7–29.8)	34.2 (32.5–36.5)	1.00
35,001–75,000	34.2 (32.7–35.7)	30.0 (28.2–31.8)	1.21 (1.02–1.44)
>75,000	37.5 (36.0–39.1)	35.5 (33.8–37.3)	1.08 (0.90–1.30)
Mother’s education			
Less than high school	11.4 (10.1–12.7)	15.3 (13.6–16.9)	1.00
High school	25.9 (24.4–27.4)	27.9 (26.0–29.8)	1.11 (0.89–1.39)
Some college	28.3 (26.9–29.7)	24.5 (22.9–26.1)	1.44 (1.16–1.79)
College graduate	34.4 (33.0–35.9)	32.4 (30.7–34.0)	1.28 (1.03–1.60)
Mother’s marital status			
Other^c^	26.5 (24.9–28.1)	31.3 (29.4–33.2)	1.00
Married	73.5 (71.9–75.1)	68.7 (66.9–70.6)	1.13 (0.98–1.31)
Mother’s age, years			
≤34	7.9 (7.0–8.8)	10.5 (9.1–11.8)	1.00
35–44	46.4 (44.8–48.1)	47.0 (45.0–48.9)	1.22 (0.99–1.51)
≥45	45.7 (44.1–47.3)	42.6 (40.7–44.5)	1.32 (1.06–1.65)
Health insurance status^d^			
Private	64.8 (63.2–66.4)	61.5 (59.5–63.5)	1.00
SCHIP or Medicaid	20.7 (19.3–22.1)	25.3 (23.3–27.2)	0.92 (0.77–1.11)
IHS or Military	1. (0.8–1.2)	1.4 (1.0–1.7)	0.73 (0.52–1.03)
Other	5.4 (4.5–6.4)	3.9 (3.1–4.7)	1.36 (1.05–1.75)
Uninsured	8.1 (7.1–9.1)	8.0 (6.8–9.2)	1.06 (0.84–1.35)
Health care provider recommended the HPV vaccine			
Yes	31.2 (29.8–32.7)	45.4 (43.5–47.3)	1.00
No	68.8 (67.3–70.3)	54.6 (52.7–56.5)	2.02 (1.82–2.53)
Survey year			
2008	22.4 (21.0–23.9)	26.4 (24.5–28.3)	1.00
2009	21.4 (19.8–22.8)	20.5 (19.0–22.0)	1.30 (1.10–1.52)
2010	21.7 (20.4–23.1)	17.4 (16.0–18.9)	1.57 (1.33–1.86)
2011	18.2 (17.1–19.3)	18.8 (17.3–20.2)	1.25 (1.06–1.47)
2012	16.3 (15.4–17.4)	17.0 (15.5–18.4)	1.26 (1.06–1.50)

^a^No-intent to initiate the HPV vaccine series in the next 12 months

^b^Includes non-Hispanic other racial/ethnic groups and biracial

^c^Never married/widowed/divorced/separated/deceased

^d^SCHIP = State Children’s Health Insurance Program; IHS = Indian Health Service

### Patterns in *no-intent to receive* the HPV vaccine series, 2008–2012

Table 4 presents patterns across survey years in parental “no-intent” for their daughters to receive the HPV vaccine series within the next 12 months, by socio-demographic characteristics. Among parents who did not receive a health care provider recommendation for their daughter to receive the HPV vaccine series, the proportion of parents who reported “no-intent” was significantly lower in survey year 2012 (35.0%, 95% CI: 32.2–37.7%) when compared to survey years 2008 (44.3%, 95% CI: (41.5–47.2%), 2009 (42.0%, 95% CI: 39.2–44.8%), and 2010 (44.9%, 95% CI: 42.2–47.6%). Additionally, there was a significant increase through survey years 2009–2012 when compared to survey year 2008 among the following factors: mothers with a college education, mothers 45 years of age or older, and higher income were all associated with an increase in the proportion of parents who reported “no-intent”.

**Table 4:** Patterns in no-intent^a^ for the HPV vaccine according to socio-demographic factors, National Immunization Survey - Teen, 2008 – 2012 (*n* = 21,467)

	Survey year				
Socio-demographic Factor	2008 (*n* = 4,605)	2009 (*n* = 4,424)	2010 (*n* = 4,173)	2011 (*n* = 4,761)	2012 (*n* = 3,504)
Age, years					
13	12.3 (10.4–14.1)	13.7 (11.8–15.5)	15.2 (13.3–17.1)	14.2 (12.5–15.9)	14.0 (12.0–15.9)
14	11.4 (9.4–13.3)	13.8 (11.8–15.8)	12.4 (10.5–14.3)	12.8 (11.1–14.4)	12.3 (10.5–14.1)
15	11.0 (9.2–12.9)	12.3 (10.7–14.0)	13.5 (11.6–15.5)	11.4 (10.0–12.9)	11.3 (9.5–13.1)
16	11.9 (10.0–13.8)	11.9 (9.4–14.3)	12.1 (10.3–13.9)	12.4 (10.5–14.2)	11.7 (9.7–13.8)
17	9.3 (7.8–10.8)	9.2 (7.9–10.5)	11.8 (10.1–13.5)	8.4 (7.2–9.5)^c^	9.6 (7.8–11.3)
Race/ethnicity					
White, non–Hispanic	33.9 (31.3–36.5)	38.4 (35.8–41.0)	39.7 (37.1–42.2)	38.2 (35.8–40.5)	35.8 (33.2–38.4)
Black, non-Hispanic	9.0 (7.5–10.5)	8.9 (7.5–10.3)	10.9 (9.2–12.6)	8.3 (6.8–9.8)	8.5 (6.6–10.3)
Hispanic	9.5 (7.4–11.6)	9.5 (7.2–11.8)	10.2 (8.0–12.3)	7.7 (6.3–9.1)	8.2 (6.5–10.0)
Other^b^	3.5 (2.5–4.4)	4.1 (3.1–5.1)	4.4 (3.4–5.4)	5.0 (3.7–6.2)	6.5 (4.8–8.1)
Annual household income, US $					
≤35,000	15.8 (13.5–18.2)	15.6 (13.5–17.7)	19.2 (16.8–21.7)	17.8 (15.8–19.9)	16.4 (13.9–18.8)
35,001–75,000	20.3 (18.1–22.5)	20.8 (18.3–23.2)	20.7 (18.7–22.8)	21.0 (18.9–23.2)	19.4 (17.1–21.6)
>75,000	19.7 (17.6–21.9)	24.6 (22.2–26.9)^e^	25.1 (22.8–27.3)^e^	20.2 (18.5–21.9)^f,g^	23.2 (20.9–25.4)
Mother’s education					
Less than high school	8.1 (6.0–10.1)	6.9 (5.3–8.5)	7.6 (5.6–9.7)	5.2 (4.0–6.4)	5.6 (3.8–7.4)
High school	15.3(13.3–17.4)	16.6 (13.9–19.3)	16.9 (14.9–18.9)	15.6(13.6–17.6)	12.5 (10.6–14.4)
Some college	15.7 (13.8–17.6)	15.9 (14.2–17.5)	18.9 (16.9–20.9)	16.5(14.7–18.2)	18.3 (16.0–20.6)
College graduate	16.8 (14.8–18.8)	21.6 (19.5–23.6)^e^	21.5 (19.4–23.6)^e^	21.9(19.9–23.8)^e^	22.5 (20.3–24.7)^e^
Mother’s marital status					
Married	42.2 (39.4–45.1)	46.7 (44.0–49.4)	48.9 (46.1–51.6)	40.9 (38.5–43.3)	40.7 (37.9–43.5)
Other^c^	13.7 (11.5–15.8)	14.2(11.7–16.7)	16.2 (14.0–18.3)	18.2 (16.0–20.4)^e^	18.2 (15.6–20.8)
Mother’s age, years					
≤34	4.5 (3.2–5.7)	3.7 (2.9–4.6)	5.5 (4.1–7.0)	5.5 (4.1–6.8)	4.4 (3.1–5.7)
35–44	28.1 (25.4–30.8)	29.1 (26.4–31.8)	28.7 (26.1–31.3)	26.6 (24.4–28.8)	25.9 (23.3–28.5)
≥45	23.3 (21.1–25.1)	28.0 (25.6–30.5)^e^	30.8 (28.4–33.2)^e^	27.0 (24.8–29.2)	28.6 (26.0–31.1)^e^
Health insurance status^d^					
Private	37.0 (34.3–39.7)	40.0 (37.4–42.7)	41.4 (38.8–44.0)	37.9 (35.5–40.4)	37.6 (34.8–40.3)
SCHIP or Medicaid	11.5 (9.4–13.5)	10.2 (8.7–11.7)	13.7 (11.6–15.9)	13.2 (11.5–14.9)	13.8 (11.4–16.1)
IHS or Military	0.4 (0.2–0.6)	0.7 (0.4–1.0)	0.8 (0.5–1.1)	0.6 (0.3–0.8)	0.6 (0.2–1.0)
Other	2.4 (1.7–3.0)	5.0 (2.9–7.1)	2.5 (1.9–3.1)	3.3 (2.3–4.2)	3.3 (2.3–4.3)
Uninsured	4.6 (3.2–6.1)	5.0 (3.6–6.3)	6.6 (4.8–8.4)	4.1 (3.2–5.1)	3.7 (2.7–4.7)
Healthcare provider recommended HPV vaccine					
Yes	11.6 (9.9–13.2)	18.9 (16.7–21.1)	20.1 (17.8–22.5)	21.5 (19.4–23.6)	24.0 (21.5–26.4)
No	44.3 (41.5–47.2)	42.0 (39.2–44.8)	44.9(42.2–47.6)	37.6 (35.2–40.0)	35.0 (32.2–37.7)^e,f,g^

^a^No-intent to initiate the HPV vaccine series in the next 12 months

^b^Includes non-Hispanic other racial/ethnic groups and biracial

^c^Never married/widowed/divorced/separated

^d^SCHIP = State Children’s Health Insurance Program; IHS = Indian Health Service

^e^Significantly different compared to the survey year 2008

^f^Significantly different compared to the survey year 2009

^g^Significantly different compared to the survey year 2010

#### Factors associated with reasons for “no-intent” to receive the HPV vaccine series

Of the 12,274 parents who reported “no-intent” for their daughters to receive the HPV vaccine series within the next 12 months, 92.3% of parents cited one reason, 6.6% cited two, and 1.1% cited three or more reasons with the maximum being seven reasons. The proportion of parents who reported one or more reasons which fall in one of more of the five domains are as follows: 43.8% v*accine misinformation,* 18.0% *safety and effective concerns, 17.0% lack of knowledge about the vaccine*, 16.0% *systemic barriers,* 5.5% *socio-cultural reasons*.

Socio-demographic factors associated with parental reasons for “no-intent” for their daughters to receive the HPV vaccine series within the next 12 months is presented in Table 5. Lack of knowledge about the vaccine as a reason for “no-intent” were significantly greater among female adolescents 15 years of age (AOR: 1.37 [95% CI: 1.05–1.80]), and 16 years of age (AOR: 1.52 [95% CI: 1.13–2.05]) compared to 13 years of age; Black non-Hispanics (AOR: 1.42 [95% CI: 1.09–1.86]), Hispanics (AOR: 1.58 [95% CI: 1.14–2.17]), and Other race/ethnicity (AOR: 1.45 [95% CI: 1.04–2.00]) compared to White non-Hispanic parents; mothers ≥45 years of age (AOR: 1.48 [95% CI: 1.00–2.22]) and 35–44 years of age (AOR: 1.56 [95% CI: 1.06–2.31]) compared to mothers (≤34 years of age. Compared to mothers with less than high school education, mothers with high school education (AOR: 1.52 [95% CI: 1.02–2.28]), some college education (AOR: 1.79 [95% CI: 1.20–2.68]), or with a graduate education (AOR: 1.84 [95% CI: 1.21–2.81]) had greater odds to report “*Safety and Effectiveness Concerns*”.

**Table 5:** Multivariable logistic regression analyses of factors associated with reasons for no-intent to receive^a^ human papillomavirus vaccine (HPV) series among unvaccinated female adolescents aged 13–17 years, National Immunization Survey - Teen, 2008–2012 (*n* = 12,274^b^)

	Adjusted odds ratio (95% confidence interval)^c^				
Factors	Safety and Effectiveness Concerns^d^	Systemic Barriers^e^	Vaccine Misinformation^f^	Lack of Knowledge about the Vaccine^g^	Socio-cultural reasons^h^
Age, years					
13	1.00	1.00	1.00	1.00	1.00
14	0.99 (0.78–1.27)	1.15 (0.86–1.52)	0.90 (0.74–1.10)	1.27 (0.95–1.70)	0.78 (0.54–1.13)
15	0.94 (0.75–1.19)	0.93 (0.69–1.24)	0.88 (0.72–1.08)	1.37 (1.05–1.80)	1.57 (1.09–2.26)
16	1.01 (0.79–1.30)	0.84 (0.63–1.13)	0.76 (0.61–0.93)	1.52 (1.13–2.05)	1.29 (0.82–2.01)
17	1.00 (0.78–1.29)	1.01 (0.76–1.36)	0.69 (0.56–0.84)	1.10 (0.83–1.47)	1.52 (1.01–2.30)
Race/ethnicity					
White, non–Hispanic	1.00	1.00	1.00	1.00	1.00
Black, non–Hispanic	0.75 (0.57–0.98)	1.05 (0.78–1.41)	0.98 (0.80–1.20)	1.42 (1.09–1.86)	1.63 (1.15–2.32)
Hispanic	0.80 (0.59–1.10)	1.02 (0.75–1.38)	0.93 (0.73–1.19)	1.58 (1.14–2.17)	1.14 (0.72–1.81)
Other^i^	0.70 (0.52–0.94)	1.39 (1.00–1.94)	0.80 (0.62–1.02)	1.45 (1.04–2.00)	0.88 (0.54–1.44)
Number of people in the household, per person over two	0.86 (0.81–0.91)	0.97 (0.91–1.04)	1.08 (1.03–1.14)	1.00 (0.92–1.09)	1.09 (0.98–1.22)
Annual household income, US $					
≤35,000	1.00	1.00	1.00	1.00	1.00
35,001–75,000	1.13 (0.88–1.47)	0.64 (0.49–0.83)	1.03 (0.84–1.27)	1.20 (0.91–1.59)	1.62 (1.04–2.55)
>75,000	1.10 (0.84–1.46)	0.62 (0.46–0.83)	1.04 (0.83–1.31)	0.95 (0.70–1.30)	1.91 (1.14–3.19)
Mother’s education					
Less than high school	1.00	1.00	1.00	1.00	1.00
High school	1.52 (1.02–2.28)	0.77 (0.52–1.13)	1.12 (0.82–1.53)	0.95 (0.66–1.38)	1.18 (0.58–2.43)
Some college	1.79 (1.20–2.68)	0.76 (0.53–1.10)	1.24 (0.91–1.68)	0.76 (0.53–1.08)	1.32 (0.67–2.59)
College graduate	1.84 (1.21–2.81)	0.82 (0.56–1.20)	1.51 (1.10–2.06)	0.48 (0.34–0.69)	1.55 (0.74–3.24)
Mother’s marital status					
Other^j^	1.00	1.00	1.00	1.00	1.00
Married	1.22 (0.98–1.52)	1.18 (0.92–1.52)	1.09 (0.88–1.34)	0.88 (0.63–1.22)	0.71 (0.51–0.98)
Mother’s age, years					
≤34	1.00	1.00	1.00	1.00	1.00
35–44	0.99 (0.71–1.36)	0.91 (0.63–1.33)	0.85 (0.64–1.13)	1.56 (1.06–2.31)	0.55 (0.31–0.98)
≥45	0.86 (0.62–1.21)	0.98 (0.67–1.43)	0.92 (0.69–1.23)	1.48 (1.00–2.22)	0.56 (0.31–1.01)
Health insurance status^k^					
Private	1.00	1.00	1.00	1.00	1.00
SCHIP or Medicaid	1.21 (0.93–1.56)	0.97 (0.73–1.30)	0.83 (0.66–1.02)	0.73 (0.54–1.00)	1.34 (0.86–2.07)
IHS or Military	1.20 (0.71–2.06)	0.76 (0.35–1.70)	0.75 (0.47–1.19)	0.83 (0.42–1.64)	1.63 (0.76–3.49)
Other	0.88 (0.63–1.22)	0.99 (0.66–1.47)	0.87 (0.64–1.19)	1.32 (0.77–2.27)	1.31 (0.74–2.34)
Uninsured	0.98 (0.69–1.38)	1.06 (0.73–1.52)	0.86 (0.65–1.15)	1.30 (0.91–1.88)	0.69 (0.38–1.25)
Healthcare provider recommended HPV vaccine					
Yes	1.00	1.00	1.00	1.00	1.00
No	0.42 (0.36–0.49)	6.84 (5.25–8.91)	1.18 (1.02–1.35)	0.86 (0.69–1.07)	0.49 (0.38–0.63)
Survey Year					
2008	1.00	1.00	1.00	1.00	1.00
2009	1.45 (1.09–1.93)	0.93 (0.70–1.24)	0.80 (0.64–0.99)	0.96 (0.71–1.30)	2.07 (1.39–3.07)
2010	2.83 (2.16–3.70)	0.93 (0.70–1.24)	0.85 (0.69–1.04)	0.63 (0.47–0.85)	0.95 (0.60–1.52)
2011	2.18 (1.68–2.83)	0.87 (0.65–1.16)	1.02 (0.83–1.25)	0.66 (0.50–0.87)	1.14 (0.75–1.72)
2012	1.45 (1.09–1.93)	1.57 (1.17–2.09)	0.67 (0.53–0.84)	0.64 (0.47–0.87)	1.47 (0.95–2.27)

^a^“no-intent” to initiate the HPV vaccine series in the next 12 months; ^b^Sample size with complete data on all the covariates in the logistic regression models

^c^Logistic regression models include all the variables listed; ^d^Safety and effectiveness concerns category included the cited reasons of safety and side effects, new vaccine, need more information, and effectiveness concerns; ^e^ *Systemic barriers* includes: health care provider not recommended, cost, vaccine not available, no doctor/obstetrician/gynecologist/doctor visit scheduled, and not a school requirement; ^f^Vaccine misinformation includes: not needed/not necessary, adolescent not sexually active, not appropriate age, and concern of increased sexual activity; ^g^ *lack of knowledge about the vaccine* included the response lack of knowledge; and ^h^ *Sociocultural reasons* included: family/parental decision, do not believe in immunizations, and against religion/orthodox

^i^Includes non-Hispanic other racial/ethnic groups and biracial; ^j^Never married/widowed/divorced/separated; ^k^SCHIP = State Children’s Health Insurance Program; IHS = Indian Health Service

Parents who did not receive a health care provider recommendation for their daughter to receive the HPV vaccine series had greater odds to report “*Systemic Barriers*” (AOR: 6.84 [95% CI: 5.25–8.91]) and/or “*Vaccine Misinformation*” (AOR: 1.18 [95% CI: 1.02–1.35]) compared to those parents of female adolescents with a provider recommendation. An increasing number of people in the household was also associated with greater odds to report vaccine misinformation (AOR: 1.08 [95% CI: 1.03–1.14]).

The odds of reporting “*Socio-cultural Barriers*” were significantly greater among female adolescents 15 years of age (AOR: 1.57 [95% CI: 1.09–2.26]) and, 17 years of age (AOR: 1.52 [95% CI: 1.01–2.30]) compared to 13 years of age; among Black non-Hispanic parents (AOR: 1.63 [95% CI: 1.15–2.32]) compared to White non-Hispanic parents; among parents who reported their annual household income as > $75,000 (AOR: 1.91 [95% CI: 1.14–3.19]), and $35,001–$75,000 (AOR: 1.62 [95% CI: 1.04–2.55]) compared to parents who reported their annual household income as ≤ $35,000. Parents in survey years 2009–2012 were more likely to report safety and effectiveness concerns compared to parents in the survey year 2008. Additionally, parents in the survey year 2012 (AOR: 1.57 [95% CI: 1.17–2.09]) had higher odds of reporting systemic barriers compared to parents in the survey year 2008.

## Discussion

This study is one of the first to identify sub-groups of parents across different socio-demographic factors who reported “no-intent” and reasons for “no-intent” for their daughters to receive the HPV vaccine series within the next 12 months in a nationally representative sample of unvaccinated female adolescents 13–17 years of age. Though the proportion of HPV vaccine uptake among female adolescents has significantly increased over time, this study found that three out of five parents of unvaccinated females reported “no-intent” to vaccinate their daughters within the next 12 months (2008: 55.1% ; 2009: 60.6% ; 2010: 64.6% ; 2011: 59.8% ; 2012: 60.3%). In addition, this study describes the patterns over time in socio-demographic factors of parents who reported “no-intent”. The study findings provide useful information to develop strategies for outreach and education geared toward health care providers whose lack of recommendation for the HPV vaccine significantly influenced parental “intent” for their daughters to receive the HPV vaccine series, to address parents’ concerns about the HPV vaccine across different socio-demographic characteristics. This may help improve the vaccine uptake among females 13–17 years of age.

Consistent with previous reports [25, 32, 39–41], provider recommendation was observed to significantly influence parental “intent” for their daughters to receive the HPV vaccine series; more than sixty percent of parents of unvaccinated females received no recommendation from their health care provider (2008: 70.1% ; 2009: 62.8% ; 2010: 63.4% ; 2011: 60.3% ; 2012: 55.9%). Among parents with “no-intent” who reported systemic barriers, 21.6% received a provider recommendation, compared to 3.9% of parents who did not receive a provider recommendation for their adolescent daughters. Among parents with “no-intent” who reported Safety and Effectiveness Concerns, 28.1% received a provider recommendation compared to 13.3% of parents who did not receive a provider recommendation for their adolescent daughters. Lastly, compared to parents in the survey year 2008, parents in the survey years 2009–2012 were significantly more likely to report “no-intent” and cite Safety and Effectiveness Concerns as their reason, yet less like to cite Lack of Knowledge about the Vaccine as a reason for “no-intent”. This may suggest that over time knowledge about the vaccine has increased, but along with that the safety and effectiveness concerns have also increased. These findings not only highlight the importance of a provider recommendation, but also the missed opportunities health care providers have to effectively communicate the health benefits of the HPV vaccine, specifically with regard to vaccine safety and effectiveness. Additionally, a provider recommendation would help to facilitate an improvement in vaccine acceptance and vaccination intentions [42–45].

In this study, mothers with a high school education or higher were more likely to report “no-intent” for their daughters to receive the HPV vaccine series within the next 12 months. Other studies have reported similar findings [28, 35, 46]. In addition, we found mothers with higher education were more likely to cite Safety and Effectiveness Concerns as a reason for “no-intent”. This highlights the importance of appropriately addressing safety and effectiveness concerns among all mothers, particularly those with higher education. Also, we found older mothers were more likely to report “no-intent” compared to younger mothers, and this result is also consistent with other studies [47, 48]. Older mothers, Black non-Hispanic, Hispanic, and parents of “Other” ethnicity were more likely to report “*Lack of Knowledge about the Vaccine*” as a reason for “no-intent”. Consistent with our finding, recent studies in Latino parents’ reported low HPV vaccine knowledge as one of the main barriers to vaccine receipt [41, 49]. Furthermore, parents of older adolescents Black non-Hispanic and parents with annual household income more than $35,000, and were more likely to cite “*Socio-cultural Barriers*”. These findings highlight the importance of disseminating health communication about HPV across various demographics.

Overall, this study found that the patterns in socio-demographic factors between proportions of parents reporting “no-intent” were similar across years 2008–2012. These patterns highlight that HPV vaccine acceptance beliefs among parents have not significantly changed over time, thereby impacting vaccine uptake among certain sub-groups of adolescents identified in this study. Considering that the HPV vaccine is intended to protect against HPV infection and pre-cancerous lesions caused by high-risk HPV types, delaying the initiation or refusing the vaccine altogether by these sub-groups of parents for their daughters is a matter of public health concern.

We acknowledge that the analysis of this study has some limitations. In the years 2008–2010, during which cell-phone-only households were increasing, the NIS-Teen sampling frame included only households that had landline telephones. For the first time in 2011, NIS-Teen utilized a dual-frame (both landline and cell-phone sampling frames) but the cellular household response rate was low compared to the landline household response rate in both 2011 and 2012. As a result, non-response and non-coverage bias may exist, even after adjusting sampling weights. Geographic region in the US, which we did not consider in our study, may be helpful to better understand and address parents’ reported reasons for “no-intent” to vaccinate their adolescent daughters [24]. Also, information about adolescent daughter being a first child or not would shed some insights into parents’ awareness of HPV vaccine. Lastly, parental no-intent to vaccinate their 13–17 years old daughters within the next 12 months might not necessarily result in not vaccinating beyond the 12-month period, as intention and action often vary.

## Conclusions

This study identified sub-groups of parents across different socio-demographic factors with “no-intent” for their daughters of 13–17 years of age to receive the HPV vaccine series within the next 12 months, and their reasons for such a decision. Developing strategies that target educational tools towards the identified sub-groups of parents about the purpose, safety, and efficacy of the HPV vaccine, and HPV infection, may help increase HPV vaccination acceptance, and initiation and completion rates among female adolescents. In addition, strategies to improve vaccine uptake should focus on developing communication tools for health care providers to better address parents’ reasons for “no-intent” in support of making recommendations for vaccinating the adolescents during their health-care encounter.

### Implications and contribution

This study identified subgroups of parents across different socio-demographics and the reported reasons for no-intent to vaccinate their daughters with the HPV vaccine. The socio-demographic characteristics of such parents with no-intent as identified in this study have implications for developing strategies to address the various concerns (reasons) reported by the parents with “no-intent” to vaccinate their adolescent daughters with HPV vaccine. Also, communication tools focused on health care providers would address the significant systemic failure due to lack of provider recommendation. These efforts may help increase HPV vaccine acceptance. Future studies should focus on the impact of strategies developed on HPV vaccine uptake among the subgroups of parents identified in this study.

## Abbreviations

AOR: Adjusted Odds Ratio
CI: Confidence Interval
FDA: Food and Drug Administration
HPV: Human papillomavirus
IHS: Indian Health Service
NIS: National Immunization Survey
SCHIP: State Children’s Health Insurance Program
STI: Sexually transmitted infection
